# Reduction of drive for thinness and body dissatisfaction in people with self-reported dysregulated eating behaviors after intermittent theta burst stimulation (iTBS) of the left dorsolateral prefrontal cortex

**DOI:** 10.3389/fnhum.2023.1108869

**Published:** 2023-03-17

**Authors:** Jennifer Barone, Massimiliamo Oliveri, Rosario Emanuele Bonaventura, Giuseppa Renata Mangano

**Affiliations:** Neuropsychology Lab, Department of Psychology, Educational Science and Human Movement, University of Palermo, Palermo, Sicily, Italy

**Keywords:** bulimia, drive for thinness, body dissatisfaction, iTBS, dorsolateral prefrontal cortex, binge-eating, dysregulated eating, EDA (electro dermal Activity)

## Abstract

**Aim:**

This study aimed to explore the effect of intermittent theta burst stimulation (iTBS) of the right and left dorsolateral prefrontal cortex (DLPFC) in people with self-reported dysregulated eating behaviors but without a diagnosis of eating disorders (EDs).

**Methods:**

Participants were randomly divided into two equivalent groups according to the side (right or left) of the hemisphere to be stimulated and they were tested before and after a single iTBS session. Outcome measurements were scores on self-report questionnaires assessing psychological dimensions related to eating behaviors (EDI-3), anxiety (STAI-Y), and tonic electrodermal activity.

**Results:**

The iTBS interfered with both psychological and neurophysiological measures. Significant variations of physiological arousal after iTBS of both the right and left DLPFC were witnessed by increased mean amplitude of non-specific skin conductance responses. With regard to the psychological measures, the iTBS on the left DLPFC significantly reduced the scores of the EDI-3 subscales drive for thinness and body dissatisfaction. Interestingly, these two scales are two of the three EDI-3 clinic scales (drive for thinness, body dissatisfaction, and bulimia) used as specific markers to assess the onset and/or maintenance of eating disorders.

**Conclusion:**

Our results show that the left DLPFC iTBS has an impact on the psychological dimensions that are risk factors for the onset of eating disorders, suggesting that an altered hemispheric asymmetry similar to that encountered in clinical populations is present in normal subjects even in the absence of clinical symptoms.

## Introduction

Dysregulated eating behaviors are characterized by frequent episodes of excessive food consumption during experiences of loss of inhibitory control and psychophysiological distress. These behaviors are common symptoms of several eating-related conditions, including binge eating disorder, anorexia nervosa (AN), and bulimia nervosa (BN). Subclinical dysregulated symptoms, such as sporadic episodes of binge eating, are common among young people and are risk factors for adverse physical and psychiatric outcomes later in life, including eating disorders (Kurth et al., 1995; Silén and Keski-Rahkonen, 2022). The identification of potential prodromes in non-clinical populations could be crucial to provide targeted prevention and intervention programs.

It has been suggested that several psychopathological dimensions predict the onset of binge eating behaviors including a drive for thinness, body image concerns (i.e., weight and shape concerns), appearance pressures, dietary restraint, impulsivity, and disinhibition (i.e., loss of control over eating, as applied to food intake) (Stice et al., 2008, 2017; Racine and Martin, 2017).

In particular, according to the dual-pathway model, elevated cultural pressure for thinness and internalization of the thin ideal promotes body dissatisfaction, which leads to unhealthy weight control behaviors and negative affect (i.e., anxiety and dysphoric mood as well as alexithymia), which in turn may result in binge eating and unhealthy compensatory behaviors (Stice, 2001).

Neurophysiological correlates of eating attitudes have been also explored showing that women who are preoccupied with food or anxious about eating exhibited the greatest sympathetic nervous system responses measured with the electrodermal activity (EDA) (Wilson and Mercer, 1990).

Structural and functional neuroimaging studies among this subclinical population suggested a pattern of hemispheric asymmetry in cortical excitability of the prefrontal areas with the left prefrontal cortex described as a key region for inhibitory control processes in overeating (Yokum et al., 2011; Val-Laillet et al., 2015; Gluck et al., 2017; Stice and Burger, 2019; Oliva et al., 2021). In particular, Dong and colleagues (Dong et al., 2016), in their fRMI study, suggest a positive correlation between the activation of the left dorsolateral prefrontal cortex (lDLPFC) and the impulse control compensation in response to a stronger reward signal among women with an elevated impulse to restrictive eating. Hare and colleagues (Hare et al., 2009) documented that different capacities in self-control are correlated with increased activity of the lDLPFC in food assumption.

A recent fMRI study by Oliva and colleagues (Oliva et al., 2019) explored the brain activity of two groups of non-clinical subjects, normal-weight individuals with and without binge eating episodes, during response inhibition tasks. Although the two groups did not differ in inhibitory efficiency in behavioral terms, their brain activity was different. Normal-weight individuals with binge eating episodes showed lower activation of the right middle frontal gyrus (MFG) and putamen and higher activation of the left MFG. This study stresses the importance to investigate both behavioral factors and neurophysiologic processes when an at-risk population is involved.

In line with this finding, an increasing number of studies employed non-invasive brain stimulation (NIBS) procedures aimed at enhancing the excitability of the dorsolateral prefrontal cortex (DLPC) or the dorsomedial prefrontal cortex (DMPFC) to modulate dysregulated eating behaviors and associated symptoms [see (Dalton et al., 2018) for review]. Repetitive transcranial magnetic stimulation (rTMS) studies documented a significant reduction of the self-reported urge to eat and binge eating in patients with bulimic-type dietary tendencies after a single session of high-frequency (i.e., 10 Hz) rTMS of the lDLPFC (Van den Eynde et al., 2010). However, studies employing multi-session of high-frequency (10–20 Hz) rTMS of the lDLPFC failed to report significant effects of rTMS on eating disorders outcomes compared to sham (Walpoth, 2008; Gay et al., 2016). The few studies employing transcranial direct current stimulation (tDCS) documented a reduction in eating disorders behavior in patients with bulimic after a single session of bilateral tDCS of the DLPFC (anode right/cathode left) (Kekic et al., 2017).

To the best of our knowledge, no previous studies investigated the effect of iTBS in subclinical populations with dysregulated eating behaviors. iTBS is an rTMS technique known to induce excitatory effects in the stimulated area (Huang et al., 2005). Compared to other NIBS techniques, iTBS induces longer lasting effects (Huang et al., 2005) and thus could be suitable in those studies aimed to modulate in a unique off-line session both the neurophysiological and the psychological dimensions. Indeed, recording neurophysiological parameters, such as the tonic electrodermal activity, as well as testing psychological dimensions through questionnaires, usually require a long administration time that may exceed the time “window” for the immediate after-effect of the NIBS. In this study, we explored the effect of iTBS on the right and left DLPFC in healthy subjects with self-reported dysregulated eating behaviors but without a diagnosis of eating disorders (EDs). Outcome measurements included sympathetic-mediated tonic electrodermal activity as an indicator of neurophysiological arousal and scores on self-report questionnaires assessing psychopathological conditions were known to be associated with dysregulated eating behaviors, such as the drive for thinness, body dissatisfaction, bulimia, interoceptive deficit, and anxiety.

We hypothesized that selective excitation of the left but not the right DLPFC, by means of iTBS, reduces the scores on those scales assessing psychopathological dimensions prominently related to dysregulated eating behaviors (i.e., drive for thinness and body dissatisfaction). In addition, we hypothesized a modulation of sympathetic-mediated tonic electrodermal activity following iTBS.

## Materials and methods

### Participants

In a screening phase, 22 adult volunteers (all females, mean age = 22.7 ± 3.8 years; mean education = 15.8 ± 1.6 years; mean body mass index = 25 ± 5.5) were selected from a sample of 71 healthy subjects (mean age = 22.1 ± 3.6 years; mean education = 14.2 ± 1.6) performing the eating attitude test (EAT 26) (Garner et al., 1982).

Inclusion criteria were as follows: age >18 years; body mass index (BMI; kg/m^2^) ranging from 18.5 to 30; and dysregulated behaviors predictive of bulimic tendencies. The presence of dysregulated behaviors was defined using the behavioral questions of the EAT-26 (Garner et al., 1982), a self-report questionnaire used to identify the eating disorders risk. In particular, the following items were considered: “I have gone on eating binges where I feel that I may not be able to stop”; “Ever made yourself sick (vomited) to control your weight or shape?”; “Ever used laxatives, diet pills, or diuretics to control your weight or shape?”; and “Have you ever been treated for an eating disorder?” Each of these items was scored on a 6-point scale ranging from 1 (never) to 6 (once a day or more). We enrolled participants reporting at least one binge eating episode (item 1) and one purging behavior (items 2–3) per month in the last 3 months without a history of eating disorders (item 4).

We adopted as a stopping rule the sample numerosity indicated by the a priori power analysis conducted using G*Power 3.1.9.4 (Faul et al., 2007). We set a minimum to medium effect size, f, of 0.25 an alpha of 0.05, with correlations among repeated measures, r, of 0.7. Results showed that a total sample of 22 participants with two equal-sized groups of n= 11 was required to achieve a power of 0.80.

All the selected 22 participants were screened for exclusion criteria for TMS (Rossi et al., 2011), and the included participants were right-handed, free of any medication, reported no history of neurologic and/or psychiatric disease, and had no pregnancy.

The study was carried out in accordance with the Code of Ethics of the World Medical Association (Declaration of Helsinki) and was approved by the Bioethics Committee of the University of Palermo (n°27/2020). Written informed consent was obtained from all subjects.

All the participants were unaware of the hypotheses of the study.

### Procedures

**Figure 1:**
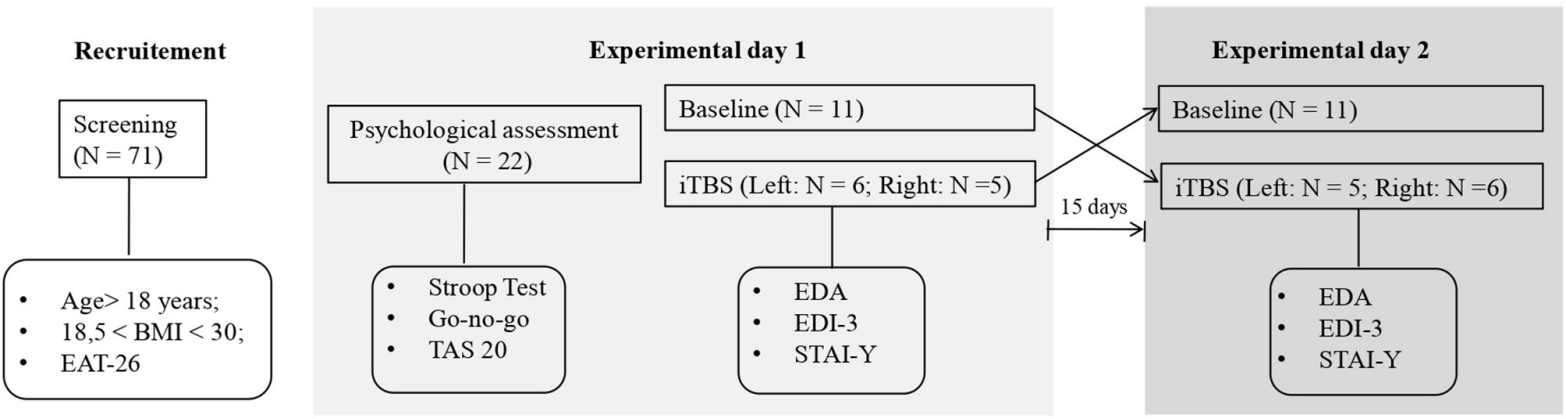
Schematic diagram of the experimental procedure.

Before the experimental conditions, participants underwent a psychological assessment conducted by a clinical psychologist to exclude the presence of alexithymia and a general deficit in cognitive control (i.e., not related to eating) (see Figure 1). The presence of alexithymia was investigated using the self-report questionnaire, Toronto Alexithymia scale-20 (TAS-20) (Bagby et al., 1994). Cognitive control was explored with regard to response inhibition and resistance to interference components (Tarantino et al., 2017) using the Italian versions of the Go no go task, from the frontal assessment battery (FAB) (Appollonio et al., 2005), and the Stroop test short-form, respectively (Caffarra et al., 2002). The Go no go task, asks participants to obey verbal commands like “tap once when I tap once” and inhibit their response (i.e., “don't tap when I tap twice”), the number and type of errors (e.g., perseverations) are computed and scored from 3 (better score) to 0 according to the norms reported in the manual of the test. In the Stroop test, which requires naming the ink color of a word while ignoring its meaning, we computed the interference effect (ColorWord – [(Word + Color)/2)]) for reaction times (in sec.) and error rates.

Participants were randomly divided into two equivalent groups according to the side (right or left) of the hemisphere to be stimulated (right DLPFC: mean age = 23 ± 5 years; mean education = 15,0.7 ± 1.7; mean BMI = 26.8 ± 6.8; left DLPFC; mean age = 22.4 ±2.2 years; mean education = 15.8 ± 1.6; mean BMI = 23.4 ± 3.4). A double-blind randomized within-subjects design was used for each group: all subjects performed a baseline session and an iTBS session on different days with a 15-day washout period. The order of each session was counterbalanced between subjects.

In the baseline session, participants underwent a battery of clinical tests assessing psychopathological conditions related to dysregulated eating behaviors, including the Italian version of the EDI-3 autoscoring (Garner, 1983) and the State-Trait Anxiety Inventory Y form (STAI-Y form) (Spielberger, 1983; Boucsein, 2012).

Eating Disorders Inventory-3 is a self-report questionnaire to measure symptoms and psychological traits in subjects with suspected or confirmed eating disorders (EDI-3). It consists of 91 items with a 5-point Likert scale. Three of the 12 primary scales, *drive for thinness* (DT), *bulimia* (B), and *body dissatisfaction* (BD), are considered eating disorder risk scales. The other nine primary scales measure psychological traits relevant to eating disorders, *low self-esteem* (LSE), *personal alienation* (PA), *interpersonal insecurity* (II), *interpersonal alienation* (IA), *interoceptive deficits* (IDs), *emotional dysregulation* (ED), *perfectionism* (P), *asceticism* (A), and *maturity fear* (MF). The qualitative classification of the profile was identified along three ranges: *non-clinical, typical clinical*, and *elevated clinical*.

State-Trait Anxiety Inventory Y form (STAI-Y form) is a self-report psychological inventory composed of 40 questions and based on a 4-point Likert scale, measuring two types of anxiety: state anxiety (STAI Y-1), about specific events, and trait anxiety (STAI Y-2), bound to personal traits.

For each self-report questionnaire, the completeness of each test's items sheets was checked, and the scoring was performed by calculating the validity scale scores, the raw scores, and the percentiles. The results were interpreted according to the Italian norms reported in the test's handbooks.

In the iTBS session, electrodermal activity (EDA) was recorded before and after stimulation, and subjects completed again the clinical tests (EDI-3 and STAI-Y) immediately after iTBS.

### Electrodermal activity (EDA)

To assess sympathetic nervous system arousal, we measured changes in tonic electrodermal activity (Boucsein, 2012). Sympathetic-mediated tonic electrodermal activity was continuously recorded at 35 Hz using the system Biopac MP36R connected with a pair of surface electrodes (6 mm diameter) positioned on the index finger contralateral to the brain stimulation site (Braithwaite et al., 2013). In each session (baseline and immediately after iTBS), we run a 5-min measurement period in the resting state, while the participants were not engaged in any task, comfortably seated in a chair in a quiet room, and instructed to be relaxed with their eyes opened. For each subject and in each session, we computed the mean amplitude of non-specific skin conductance responses (NS-SCRs). All the NS.SCRs >0.02 μS were counted and used in data analysis.

### iTBS protocol

An intermittent active TBS was applied over right and left DLPFC sites corresponding to F4 and F3 positions on the 10–20 EEG system using a Magstim Rapid 2 magnetic stimulator and figure-8 coil (70 mm). The figure-of-eight coil was applied tangentially on the target scalp site, with the handle pointing posteriorly, so as to induce a current with a posterior-to-anterior direction in the underlying brain areas. The coil was maintained on the target scalp with the same orientation across sessions using a mechanical arm and its position was monitored using a SofTaxic Navigator system (EMS). We administered the stimulation protocol originally described by Huang et al. (2005).

Stimulation paradigm parameters were as follows: frequency 50 Hz; the number of pulses 3; the number of bursts 10; cycle time 8 s; the number of cycles 20; burst frequency 5 Hz; and the total number of pulses 600. The stimulation intensity was kept at 30% of the maximal stimulator output, due to limitations in the maximal TBS intensity of the stimulator. We chose to administer equal intensity for all participants based on previous findings documenting that adjusting stimulation intensities in each subject to the individual motor threshold does not necessarily lead to more prominent effects (Kaminski et al., 2011).

### Data analyses

Normality assumptions were checked by evaluating skewness and kurtosis values documenting a normal distribution for all data (z-scores for either skewness or kurtosis < 1.96) (Kim, 2013).

First, we analyzed the data collected in the baseline session. To characterize the phenotype of the participants, a descriptive analysis of the behavioral, neurophysiological, and psychological characteristics of the sample was calculated. To explore the association of their characteristics, correlation analysis between BMI, cognitive control tasks scores, mean NS.SCRs amplitude (μS), TAS-20, STAI-Y, and EDI-3 scale scores were carried out with *Pearson's* coefficient ϱ. Bonferroni correction for multiple comparisons was applied to *p*-values. Unpaired two-tailed *t*-tests were carried out on the baseline data to determine if the two groups of participants (i.e., left and right) were similar in terms of age, education, BMI, mean NS.SCRs amplitude, and psychological tests scores.

Second, to investigate the effects of the excitatory iTBS protocol on the neurophysiological variable, repeated measure ANOVA was carried out on the mean NS.SCRs amplitude (μS), with a group (left vs. right) as a between-subjects factor, and a session (baseline vs. post-iTBS) as a within-subjects-factor.

Finally, to investigate the effects of the excitatory iTBS protocol on psychological variables two mixed MANOVAs were conducted, with one featuring the EDI-3 scores as dependent variables and the other featuring the STAI-Y scores as dependent variables. These models included *group* (left DLPFC, right DLPFC) as a between-subject factor and *session* (baseline, post-iTBS) as a within-subject factor. Following Wang and colleagues (Wang et al., 2019), the analysis of the EDI-3 scores was focused only on the subscales of drive for thinness, bulimia, body dissatisfaction, and interoceptive deficit, since they are more directly relevant to bulimia nervosa than the other subscales. We then performed repeated measures ANOVA with simple planned contrasts between baseline and iTBS sessions.

All analyses were run using the software package SPSS Statistics 23 (IBM Corp, 2021), and for all of them, the level of significance was set at *p* = 0.05.

We carried out an effect-size sensitivity analysis to determine whether the analyses were powered to detect meaningful effect sizes. Given our sample size (*n* = 22) and desired power (80%), we obtained an effect size of f = 0.65 (this corresponds to a ηp^2^ = 0.30).

## Results

### Descriptives

All participants showed normal TAS-20 scores (mean = 47.6 ± 14.1; cutoff > 61) and in both cognitive control tasks (Go no go task, mean scores = 3 ± 0; Stroop test mean interference time scores = 9.1 ± 5.6; Stroop test mean interference error scores = 0.4 ± 0.7), so we could exclude the presence of clinically relevant symptoms in affective-emotional regulation and cognitive control in our sample. The mean scores for the anxiety scales were 54.1 ± 23.4 and 58.5 ± 28.6 for STAI-1 state anxiety and STAI-2 trait anxiety, respectively, falling into the moderate anxiety range (cutoff = 40). As for EDI-3 scales, all participants showed dysregulated behaviors predictive of bulimia nervosa (BN) and anorexia with binging and purging (AN-bp) defined according to the norms reported in the manual of the test. In particular, 55%, 77%, 45%, and 45% of the sample showed clinical range (cutoff = 50° to 85° percentiles) on the following EDI-3 scales: drive for thinness, bulimia, body dissatisfaction, and perfectionism, respectively. In addition, 14% of the sample showed elevated clinical range (cutoff = 85° to 99° percentiles) on the drive for thinness and bulimia scales, and 23% of the sample showed elevated clinical range on body dissatisfaction and perfectionism scales.

Unpaired two-tailed *t*-tests revealed that the two groups of participants (i.e., left and right) were similar in terms of age, education, BMI, EDA, and psychological test variables in the baseline session (see Table 1).

**Table 1:** Characteristics of the two groups of participants.

**Characteristics**			**Left Group (n = 11)**	**Right Group (n = 11)**	**t**	***p*-value**
			**Mean (sd)**	**Mean (sd)**		
Age (years)			22.4 (2.2)	23 (5)	0.37	0.7
Education (years)			15.8 (1.6)	15.77 (1.78)	1.17	0.25
BMI (kg/m2)			23.4 (3.4)	26.8 (6.8)	1.5	0.16
Electodermal activity	Mean NS-SCRs amplitude (μS)		14.5 (6.6)	10.81 (6.29)	−1.25	0.22
Cognitive control	Go no go	Score correct response	3 (0)	3 (0)		
	Stroop Test	IE time (sec.)	8.8 (4.4)	9.5 (6.8)	0.3	0.76
		IE errors	0.36 (0.66)	0.45 (0.85)	0.27	0.78
Alexithymia	TAS-20		48.3(17)	46.9 (11.2)	−0.23	0.81
Anxiety	State (STAI Y-1)		49 (27)	59.2 (18.7)	1.02	0.32
	Trait (STAI Y-2)		52.4 (26.4)	64.7 (30.7)	1	0.32
Eating disorders inventory (EDI-3)	Drive for thinness		56.3 (28.8)	63.9 (21.3)	0.69	0.49
	Bulimia		64.9 (16.2)	69.6 (17.3)	0.66	0.51
	Body dissatisfaction		56 (26)	63.6 (28.5)	0.65	0.51
	Interoceptive deficit		43.2 (31.2)	54.1 (23.5)	0.92	0.36
	Low self-esteem		53.4 (18.1)	54.5 (23.3)	1.22	0.9
	Personal alienation		35.8 (26.7)	58.3 (28.1)	1.92	0.06
	Interpersonal insecurity		39.7 (23.3)	48.1 (28.7)	0.75	0.45
	Interpersonal alienation		39.1 (27.5)	57.8 (30.7)	1.49	1.15
	Emotional dysregulation		51.2 (26.9)	53.2(33.2)	1.15	0.87
	Perfectionism		52.6 (28.4)	71.1 (25)	1.62	0.12
	Asceticism		55.5 (25.5)	57.2 (25.4)	0.15	0.87
	Maturity fear		59.6 (28.7)	57.3 (30.3)	−0.18	0.85

BMI, body mass index; IE, Interference effect (ColorWord – [(Word + Color)/2)]); TAS-20, Toronto Alexithymia scale-20; STAI-Y, State-Trait Anxiety Inventory Y form; EDI-3, Eating Disorders Inventory-3.

### Correlations in the baseline session

Significant positive correlations were found between the BMI and the EDI-3 subscale body dissatisfaction [*r* (20) = 0.73; *p* < 0.00]. Significant negative correlations were found between the mean NS-SCRs amplitude (μS) and the EDI-3 subscale perfectionism [*r* (20) = −0.62; *p* = 0.002].

The STAI-2 subscale (trait anxiety) was positively correlated with the following EDI-3 subscales: low self-esteem (LSE) [*r* (20) = 0.66; *p* = 0.001] and personal alienation [*r* (20) = 0.71; *p* < 0.00].

Finally, the TAS-20 positively correlates with the following EDI-3 subscales: personal alienation [*r* (20) = 0.56; *p* = 0.006] and interoceptive deficit [*r* (20) = 0.67; *p* = 0.0001].

### Measures post-iTBS

Descriptive statistics of each administered test for the two groups of participants before and after the DLPFC iTBS are shown in Table 2.

**Table 2:** Descriptive statistics of each administered test for the two groups of participants before and after the DLPFC iTBS.

**Characteristics**		**Left Group (*****n*** = **11) Mean (sd)**		**Right Group (*****n*** = **11) Mean (sd)**	
		**Baseline**	**Post Left iTBS**	**Baseline**	**Post Right iTBS**
Electodermal activity	Mean NS-SCRs amplitude (μS)	14 (6)	17 (7)	11 (5)	13 (6)
Anxiety	State (STAI Y-1)	49 (27)	42 (20.9)	59.2 (18.7)	51 (23)
	Trait (STAI Y-2)	52.4 (26.4)	57 (24.3)	64.7 (30.7)	67 (25)
Eating Disorders Inventory (EDI-3)	Drive for thinness	56.3 (28.8)	49 (29)	63.9 (21.3)	65 (21)
	Bulimia	64.9 (16.2)	53 (27)	69.6 (17.3)	56 (32)
	Body dissatisfaction	56 (26)	52 (28)	63.6 (28.5)	66 (29)
	Interoceptive deficit	43.2 (31.2)	43 (32)	54.1 (23.5)	44 (30)
	Low self-esteem	53.4 (18.1)	48 (23)	54.5 (23.3)	57 (24)
	Personal alienation	35.8 (26.7)	36 (25)	58.3 (28.1)	56 (31)
	Interpersonal insecurity	39.7 (23.3)	37 (30)	48.1 (28.7)	47 (28)
	Interpersonal alienation	39.1 (27.5)	35 (34)	57.8 (30.7)	54 (28)
	Emotional dysregulation	51.2 (26.9)	49 (36)	53.2(33.2)	49 (30)
	Perfectionism	52.6 (28.4)	61 (24)	71.1 (25)	66 (29)
	Asceticism	55.5 (25.5)	55 (30)	57.2 (25.4)	56 (29)
	Maturity fear	59.6 (28.7)	61 8 (23)	57.3 (30.3)	61 (28)

NS-SCRs, non-specific skin conductance responses; STAI-Y, State-Trait Anxiety Inventory Y form; EDI-3, Eating Disorders Inventory-3.

### Electrodermal activity

The ANOVA conducted on the mean NS-SCRs amplitude (μS) showed a significant main effect of the factor Session [*F*_(1, 20)_ = 25,7, *p* = 0.00005, ηp^2^ = 0.56]. Independently of the group (i.e., left or right), an increase in the mean NS-SCRs amplitude was found following iTBS [13.1 μS vs. 15.5 μS, *p* = 0.00005]. No other significant main effects of Group [*F*_(1, 20)_ = 1,44 *p* = 0.24, ηp^2^ = 0.06] as well interaction Session x Group were found [*F*_(1, 20)_ = 0.06 *p* = 0.79, ηp^2^ = 0.003].

### EDI-3 subscales

**Figure 2:**
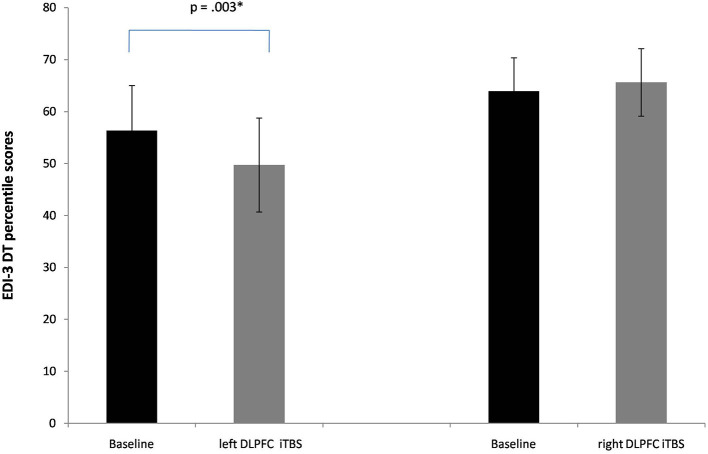
Baseline and post-iTBS percentile scores in the EDI-3 subscale drive for thinness (DT). Error bars indicate standard error. *p < 0.05.

The MANOVA showed a significant interaction between Session x Group [Pillai's trace = 0.53, *F*_(4, 17)_ = 4.78, *p* = 0.009, ηp^2^ = 0.53]. No significant main effects of Group [Pillai's trace = 0.05, *F*_(4, 17)_ = 0.18, *p* = 0.94, ηp^2^ = 0.05] or Session [Pillai's trace = 0.36, *F*_(4, 17)_ = 2.47, *p* = 0.08, ηp^2^ = 0.36] were found. Separate follow-up univariate ANOVAs revealed a significant interaction between Session X Group in the subscale drive for thinness [*F*_(1, 20)_ = 8.7, *p* = 0.008, ηp^2^ = 0.30]. In particular, a significant reduction of these subscale's scores was found only when iTBS was applied on the lDLPFC [56, 3 vs. 49,7; *p* = 0.003, see Figure 2].

**Figure 3:**
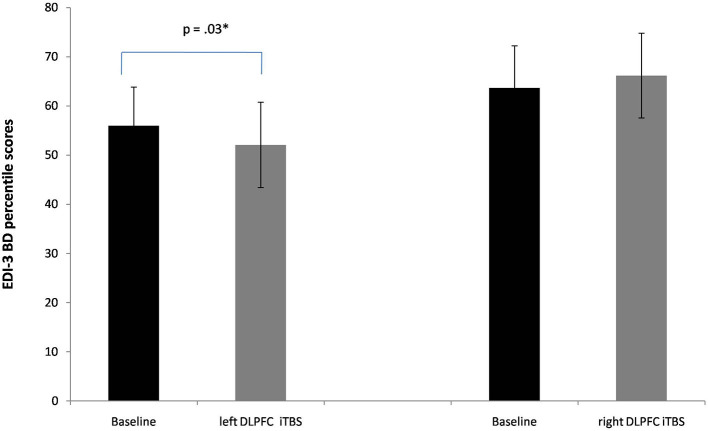
Baseline and post-iTBS percentile scores in the EDI-3 subscale body dissatisfaction (BD). Error bars indicate standard error. *p < 0.05.

A significant interaction between Session X Group was found also in the subscale body dissatisfaction [*F*_(1, 20)_ = 7.7, *p* = 0.01, ηp^2^ = 0.27]. Again, a significant reduction of this subscale's scores was found when iTBS was applied to the lDLPFC [56 vs. 52; *p* = 0.03, see Figure 3].

Independently of the group, significant main effects for the factor Session were found in the subscale bulimia [*F*_(1, 20)_ = 6.1; *p* = 0.02, ηp^2^ = 0.23]. A significant reduction of this subscale's scores was found after iTBS [67.2 vs. 55; *p* = 0.02].

There were no other significant main effects or interactions (see Table 3).

**Table 3:** Univariate ANOVAs.

	**Subscale**	**Effects**	***F* (df)**	***p*-value**	**η^2^**
EDI-3	Drive for thinness	Group	1.16 (1.20)	0.29	0.05
		Session	3.02 (1.20)	0.09	0.13
		Session*Group	8.7 (1.20)	0.008	0.30
	Bulimia	Group	0.15 (1.20)	0.70	0.008
		Session	6.1 (1.20)	0.02	0.23
		Session*Group	0.05 (1.20)	0.81	0.003
	Body dissatisfaction	Group	0.83 (1.20)	0.70	0.04
		Session	0.33 (1.20)	0.57	0.01
		Session*Group	7.7 (1.20)	0.01	0.27
	Interoceptive deficits	Group	0.24 (1.20)	0.62	0.01
		Session	2.04 (1.20)	0.16	0.09
		Session*Group	2.39 (1.20)	0.13	0.10
STAI-Y	State anxiety	Group	1.46 (1.20)	0.24	0.06
		Session	1.9 (1.20)	0.18	0.08
		Session*Group	0.005 (1.20)	0.94	0.0001
	Trait anxiety	Group	0.95 (1.20)	0.34	0.04
		Session	1.54 (1.20)	0.22	0.07
		Session*Group	0.22 (1.20)	0.64	0.01

### STAI-Y

The MANOVA showed a significant main effect of Session [Pillai's trace = 0.27, *F*_(2, 19)_ = 3.65, *p* = 0.04, ηp^2^ = 0.27]. No other significant main effects of Group [Pillai's trace = 0.08, *F*_(2, 19)_ = 0.70, *p* = 0.51, ηp^2^ = 0.08], as well interactions Session x Group [Pillai's trace = 0.01, *F*_(2, 19)_ = 0.13, *p* = 0.87, ηp^2^ = 0.01], were found.

Separate follow-up univariate ANOVAs revealed no significant main effects as well as interactions either in STAI-1 state anxiety or in STAI-2 trait anxiety (see Table 3).

## Discussion

The present study explored the effects of a single session of iTBS over the right and left DLPFC in a non-clinical population of individuals with self-reported dysregulated eating behaviors (indexed by the behavioral questions of the EAT 26) and psychological dimensions associated (indexed by the EDI-3 questionary) using neurophysiological and psychological outcome measures.

A preliminary correlational analysis of the characteristics of our sample collected in baseline session (i.e., before application of iTBS of the DLPFC), revealed that several psychological dimensions of eating disorders were significantly correlated with BMI (i.e., body dissatisfaction), mean NS-SCRs amplitude (i.e., perfectionism), trait anxiety (i.e., personal alienation, low self-esteem), and alexithymic traits (i.e., personal alienation, interoceptive deficit), confirming the well-known multifactorial nature of dysregulated eating behaviors (Wilson and Mercer, 1990; Stice, 2001; Stice and Whitenton, 2002; Stice et al., 2008, 2017; Racine and Martin, 2017).

The main result of our study is that iTBS interfered with both neurophysiological and psychological measures. Significantly variations of physiological arousal after iTBS of both the right and left DLPFC were witnessed by increased mean amplitude of non-specific skin conductance responses. We interpret these effects as the result of a non-specific arousal activation induced by brain stimulation, rather than as correlated to a specific modulation of the vegetative system. Indeed, previous studies (Bracco et al., 2017) showed a direct correlation between sympathetic activation with right hemispheric activation, while a negative correlation was found with left hemispheric activation.

As concerns to the psychological measures, we found that the iTBS interferes with all three EDI-3 clinic scales (DT, B, and BD) used as specific markers to assess the onset and/or maintenance of eating disorders (Garner, 1983; Lowe et al., 2007). In particular, the iTBS over the left (but not the right) DLPFC significantly reduced the scores of the EDI-3 scales drive for thinness and body dissatisfaction. Both these constructs are core features in different subtypes of eating disorders (e.g., bulimia nervosa, Anorexia-binge eating/purging type, anorexia-restrictive) when body dissatisfaction refers to negative evaluations of one's body, such as shape, size, and weight is primarily a function of the obsession with thinness and/or fear of weight gain (Stice, 2001).

Our findings are in line with previous neuroimaging (Hare et al., 2009; Yokum et al., 2011; Val-Laillet et al., 2015; Dong et al., 2016; Gluck et al., 2017; Oliva et al., 2019; Stice and Burger, 2019) and high-frequency rTMS studies [see (Dalton et al., 2018) for review], suggesting a sub-activation of the left DLPFC in dysregulated eating behaviors.

Since our sample included individuals with psychological dimensions associated with dysregulated eating behavior but no diagnosis of ED, our results suggest that an altered hemispheric asymmetry, similar to that encountered in clinical populations, exists in healthy subjects even in the absence of clinical symptoms.

We found a significant reduction in the Bulimia scale scores after iTBS of both the right and left DLPFC. It is noteworthy that the bulimia construct assesses the tendency to think about and engage in bouts of uncontrollable overeating (e.g., “I eat when I am upset”), in other words, the habitual presence of binging behaviors. Such behavioral modulations are difficult to interpret after a single session of stimulation. To better understand the specific effect of the left and right DLPFC on the frequency of bulimic behaviors, future studies including multiple iTBS sessions and follow-ups are needed.

A lack of iTBS effects was found in the scales investigating anxiety. Although our sample falls into the moderate anxiety range for both state anxiety and trait anxiety, we did not find any variations in the scores after iTBS. Indeed, the few studies that applied excitatory stimulation protocols in anxiety disorders found that when the DLPFC was targeted and multiple sessions were employed, clinical symptoms decreased, implying that evidence is needed to strengthen conclusions about the effectiveness of a single session of NIBS in anxiety disorders (Vergallito et al., 2021).

The present study has some limitations that could be addressed in future research. As noted earlier, here we focused on the psychological dimensions associated with dysregulated eating disorders and we did not control for change also in bulimic behaviors in a follow-up. Second, although not essential for the purpose of our study, which aimed to test the effect of iTBS over the left DLPFC compared to a control site as the right DLPFC, we did not control for a sham group or condition.

In conclusion, our findings suggested an impact of left DLPFC iTBS on the psychological dimensions that represent risk factors for the onset of dysregulated eating disorders. Knowledge of this issue could be useful to put asymmetric cortical excitability in the frontal area as a potential neurophysiological marker of dysregulated eating along with a continuum between normality and disease in order to prevent ED.

## Data availability statement

The raw data supporting the conclusions of this article will be made available by the authors, without undue reservation.

## Ethics statement

The studies involving human participants were reviewed and approved by Comitato di Bioetica Università degli Studi di Palermo. The patients/participants provided their written informed consent to participate in this study.

## Author contributions

JB, MO, and GM designed the research and wrote the paper. JB and RB collected the data. MO and GM analyzed and discussed the data. All authors contributed to the article and approved the submitted version.
